# Physician-staffed HEMS: triage accuracy beyond diagnostic agreement in paediatric trauma

**DOI:** 10.1186/s13049-026-01706-1

**Published:** 2026-10-01

**Authors:** Urs Pietsch, Julian Klug, Christian P. Both, Alexander Fuchs

**Affiliations:** 1 Division of Perioperative Intensive Care Medicine, Cantonal Hospital St. Gallen, St. Gallen, Switzerland; 2 Swiss Air-Rescue, Schweizerische Rettungsflugwacht Rega, Zurich, Switzerland; 3 Department of Emergency Medicine, Bern University Hospital, Inselspital, University of Bern, Bern, Switzerland; 4 Department of Intensive Care Medicine, Lausanne University Hospital, Lausanne, Switzerland; 5 Children’s University Hospital Zurich, Zurich, Switzerland; 6 Department of Anaesthesia, Children’s Hospital of Eastern Switzerland, St. Gallen, Switzerland; 7 Department of Anaesthesiology and Pain Medicine, Bern University Hospital, Inselspital, University of Bern, Freiburgstrasse, Bern, 3010 Switzerland

We thank the correspondents [1] for their thoughtful comments on our study [2], and welcome the opportunity to clarify several points.

We agree that Cohen’s kappa quantifies chance-corrected agreement rather than clinically weighted diagnostic performance. This measure can coexist with prevalence-dependent misclassification of rarer, high-consequence categories, as described in the methodological literature on agreement paradoxes [3, 4]. We share the correspondents’ view that the overall kappa of 0.64 should not be treated as a standalone measure of triage accuracy.

We would clarify the basis of our conclusion. Our statement that the findings support *“the clinical value of HEMS physicians to correctly triage paediatric patients”* was not based solely on diagnostic agreement. Rather, it reflected several factors in our study. A physician-assessed NACA ≥ 5 identified in-hospital death with a sensitivity of 1.0 and a specificity of 0.942, meaning this threshold missed no fatalities in our cohort. Higher NACA scores correlated with in-hospital ISS (Spearman 0.52) and use of intraosseous access (OR 3.93), and were inversely associated with hospital survival (OR 0.015). By inclusion criterion, all patients in our cohort were transported to one of three major paediatric trauma centres, so destination-level triage was appropriate by design. Board-certified anaesthetists performed clinical assessments and advanced prehospital interventions on scene, including tracheal intubation, as part of the physician-staffed HEMS model [5].

Regarding diagnosis-specific measures, we would highlight that Fig. 3 reportedin the original manuscript reports diagnostic accuracy by category rather than the aggregate kappa alone, indicating that polytrauma was underdiagnosed, whereas traumatic brain injury and extremity/pelvic injuries were overdiagnosed [2]. Figure 4 reported in the original manuscript, the Sankey diagram of diagnostic trajectories from injury mechanism through prehospital and in-hospital diagnosis to outcome, illustrates the same pattern, with polytrauma and burns showing the most pronounced misclassification [2].

We report diagnostic accuracy using corresponding in-hospital diagnoses as the reference standard. For each diagnosis, we calculated sensitivity, specificity, positive predictive value, negative predictive value, and accuracy from patient-level one-versus-rest contingency tables. We estimated 95% confidence intervals using the Wilson score method. Table 1 includes trauma categories with ≥ 20 available in-hospital and prehospital diagnoses.

**Table 1:** Diagnostic performance by trauma category (categories with ≥ 20 in-hospital and ≥ 20 prehospital diagnoses)

Diagnosis	In-hospital diagnosis *n*	Pre-hospital positive *n*	TP	FP	FN	TN	Sensitivity (95% CI)	Specificity (95% CI)	PPV (95% CI)	NPV (95% CI)	Accuracy (95% CI)
Traumatic brain or cervical spine injury	97	109	90	19	7	149	92.8% (85.8–96.5%)	88.7% (83.0–92.6%)	82.6% (74.4–88.5%)	95.5% (91.0–97.8%)	90.2% (86.0–93.2%)
Chest trauma	29	26	8	18	21	218	27.6% (14.7–45.7%)	92.4% (88.3–95.1%)	30.8% (16.5–50.0%)	91.2% (86.9–94.2%)	85.3% (80.5–89.0%)
Pelvic trauma / lumbar spine	21	30	12	18	9	226	57.1% (36.5–75.5%)	92.6% (88.6–95.3%)	40.0% (24.6–57.7%)	96.2% (92.9–98.0%)	89.8% (85.6–92.9%)
Upper extremity trauma	47	40	28	12	19	206	59.6% (45.3–72.4%)	94.5% (90.6–96.8%)	70.0% (54.6–81.9%)	91.6% (87.2–94.5%)	88.3% (83.9–91.6%)
Lower extremity trauma	57	57	45	12	12	196	78.9% (66.7–87.5%)	94.2% (90.2–96.7%)	78.9% (66.7–87.5%)	94.2% (90.2–96.7%)	90.9% (86.9–93.8%)

Abbreviations: TP, true positive; FP, false positive; FN, false negative; TN, true negative; PPV, positive predictive value; NPV, negative predictive value

We deliberately refrained, however, from reporting class-specific sensitivity, specificity, or predictive values for polytrauma, because only 27 of 272 hospitalised patients (9.9%) carried this diagnosis, and only a small fraction of these had a granular, injury-level breakdown. Point estimates from such small numbers would carry very wide confidence intervals and risk conveying false precision rather than clarifying the false-negative rate the correspondents rightly seek. We view this as a genuine data limitation rather than an oversight, consistent with our discussion of anchoring on a single dominant injury, such as traumatic brain injury, as a plausible mechanism for missed concurrent injuries.

We also agree that overdiagnosis of traumatic brain injury and extremity/pelvic injury may reflect deliberate, safety-oriented overtriage rather than error, consistent with established field-triage guidance accepting a controlled overtriage rate to minimise undertriage [6, 7].

We are grateful for this correspondence, which sharpens the distinction between diagnostic agreement and overall prehospital triage accuracy for future research.

## Data Availability

The data that support the findings of this study are available from the corresponding author with a reasonable research question and approval of the responsible Swiss Ethics Committee.
